# RNA Binding Proteins as Pioneer Determinants of Infection: Protective, Proviral, or Both?

**DOI:** 10.3390/v13112172

**Published:** 2021-10-28

**Authors:** Samantha Lisy, Katherine Rothamel, Manuel Ascano

**Affiliations:** 1 Department of Biochemistry, Vanderbilt University School of Medicine, Nashville, TN 37232, USA; samantha.m.lisy@vanderbilt.edu (S.L.); krothamel@ucsd.edu (K.R.); 2 Department of Cellular and Molecular Medicine, University of California San Diego, La Jolla, CA 92037, USA

**Keywords:** RNA binding proteins, innate immunity, viral infection, host vs. pathogen, post-transcriptional gene regulation, RNA sensing

## Abstract

As the first intracellular host factors that directly interact with the genomes of RNA viruses, RNA binding proteins (RBPs) have a profound impact on the outcome of an infection. Recent discoveries brought about by new methodologies have led to an unprecedented ability to peer into the earliest events between viral RNA and the RBPs that act upon them. These discoveries have sparked a re-evaluation of current paradigms surrounding RBPs and post-transcriptional gene regulation. Here, we highlight questions that have bloomed from the implementation of these novel approaches. Canonical RBPs can impact the fates of both cellular and viral RNA during infection, sometimes in conflicting ways. Noncanonical RBPs, some of which were first characterized via interactions with viral RNA, may encompass physiological roles beyond viral pathogenesis. We discuss how these RBPs might discriminate between an RNA of either cellular or viral origin and thus exert either pro- or antiviral effects—which is a particular challenge as viruses contain mechanisms to mimic molecular features of cellular RNA.

## 1. Introduction

In the simplest terms, an RNA binding protein is a protein that can directly associate with RNA. However, upon recognition of a cognate RNA target, these proteins are responsible for a wide array of essential biological functions; from the post-transcriptional gene regulation (PTGR) of cellular transcripts to acting as sentinels for the detection of aberrant RNA, a potential sign of cellular damage or pathogen invasion. With respect to the innate immune system, one can broadly consider proteins that bind to RNA as belonging to two major categories: (1) The RNA-specific subset of pattern recognition receptors (PRRs) responsible for sensing pathogen-associated or damage-associated molecular patterns (PAMPs, DAMPs) and eliciting immune signaling pathways, or (2) as the more classically defined RNA binding proteins (RBPs) that manage gene expression at the level of RNA. While there exist many immune potent ligands that are of a firm pathogenic origin (e.g., prokaryotic-specific lipopolysaccharides), appropriate detection of an RNA as foreign is less straightforward. As an RNA virus infects a cell, viral RNA (vRNA) is released into a milieu full of cellular RNA. Consequently, specialized RBPs must be able to discriminate whether an RNA is truly host derived. Although significant efforts have been made into defining features of “self” vs. “foreign” RNA, how—and whether—these marks can strictly distinguish the identity of an RNA is less clear [1,2,3]. Viruses can acquire molecular features of cellular RNA. This in turn allows a virus to evade the PRR and/or RBP-dependent immune responses. We will begin this review by first providing a brief overview of how RNA-specific PRRs sense and elicit signal transduction events to activate the innate immune system. These signal transduction events can lead to profound transcriptional changes that convert a recently infected cell to a more pro-inflammatory and antiviral state. During this punctuated orchestration of new gene expression, the roles of RBPs as the principal effectors of PTGR in innate immunity become more prominent. Thus, we will spend the rest of the review discussing our current understanding of how RBPs facilitate PTGR in the face of a dynamically changing transcriptome–and therefore changing substrate pool, all while there remains the potential that some of those transcripts can be of pathogenic origin.

## 2. RNA Recognition by Host Proteins and Pattern Recognition Receptors

RNA recognition is primarily driven by three non-exclusive molecular features: availability, localization, and sequence/structure [2]. The availability of an RNA is influenced by the shielding of sequence/structural elements that can preclude the binding of other RBPs to the RNA. Localization of an RNA can be defined by whether an RNA is restricted to a particular subcellular location; principally, whether it exists in the same space as cognate RBPs. Sequence-dependent recognition of an RNA by RBPs can occur through conserved RNA binding domains (RBDs) encoded within the RBP–most of which typically recognize relatively short nucleotide sequences or possess more simplified pattern-driven preferences, such as purine-rich or pyrimidine-rich tracts of RNA [4,5,6]. The RNA Recognition Motif (RRM), which binds to AU-rich elements (AREs) is one of the most common RBDs. RNA-intrinsic features that affect its recognition by an RBP include the propensity of an RNA to form secondary structures, the length of the RNA, and modifications such as the 5′ cap, the polyA tail, and internal base modifications such as N-methyl-6-adenosine (m6A)) [2,3].

**Figure 1:**
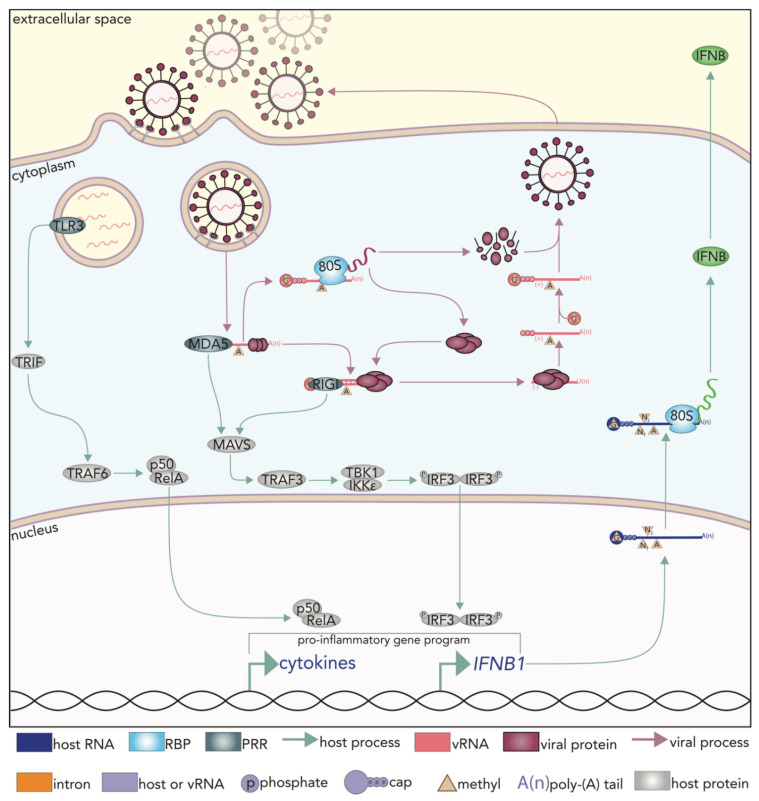
PRR detection of viral RNA initiates a signaling cascade that causes widespread transcriptomic changes to cells. RNA-specific pattern recognition receptors (PRRs), some of which are pictured here, are responsible for detecting viral RNA. PRRs elicit a signaling cascade that typically results in a type I interferon response with the release of interferon beta (IFNB) into the extracellular space. Modelled is the Chikungunya virus replication cycle [10]. Toll like receptor-3 (TLR3) detects endosomal RNA Melanoma differentiation-associated protein (MDA5) and Retinoic acid inducible gene (RIG-I) detect cytoplasmic RNA. Activation of TLR3 results in Toll/Interluekin-1 receptor domain containing adapter-inducing IFNB (TRIF)-mediated induction of the IFNB and NF-κB (p50/RelA) pathways. Activation of the RLRs induces an Interferon regulatory factor 3 (IRF3)-mediated signal transduction response that results in the expression of *IFNB1*. Innate immune activation of cells via NF-κB, IRF activities, or a type I interferon stimulation lead to profound transcriptomic changes for which existing and newly expressed RBPs must engage in order to properly orchestrate an effective pro-inflammatory and anti-viral response.

While relatively more specific molecular features can be attributed to RNA recognition by classical RBPs, the PRRs that recognize RNA mainly participate in non-sequence specific interactions. Toll-like receptors (TLRs) are among the RNA-sensing PRRs and three of which (TLRs -3, -7, and -8) have demonstrated RNA binding capabilities [1,2,3]. While TLR3 recognizes double stranded RNA (dsRNA) longer than 35 bp, TLRs -7 and -8 bind to single stranded RNA (ssRNA) [2,7,8,9]. Nevertheless, the principal criterion for all TLRs to recognize RNA is the location of the RNA [1,2,3,10]. TLRs -3, -7, and -8 are localized on the membranes of endocytic vesicles and are thus able to recognize endosomal RNA as a potential PAMP [1,2,3,10]. Notwithstanding the features by which RNA is recognized, the primary purpose of the RNA-sensing TLRs is to initiate an immune signaling cascade upon detection of aberrant RNA. While there are differences in how a PRR signal is elicited, activation of TLRs -3, -7, or -8 results in the stimulation of the NF-κB pathway and/or activation of the transcription factors Interferon regulatory factor 3 (IRF3), Interferon regulatory factor 5 (IRF5), and Interferon regulatory factor 7 (IRF7) (Figure 1) [2,10,11].

The second prominent class of PRRs that can detect RNAs are the Retinoic acid inducible gene (RIG-I, or DDX58)-Like Receptors (RLRs). RLRs are a family of cytosolic PRRs comprised of RIG-I, Melanoma differentiation-association protein 5 (MDA5) and Laboratory of genetics and physiology 2 (LGP2) [11,12]. These receptors contain two conserved RNA helicase domains responsible for RNA sensing, and one of which consists of a DExD/H-box helicase [11]. RIG-I recognizes short (10–300 bp) blunt-ended duplexed RNA with either 5′-diphosphate (PP) or -triphosphate (PPP) overhangs [11,12]. RIG-I can also detect single stranded RNA with 5′PPP overhangs [11]. Notably, RNA must also be unmethylated at the 2′ hydroxyl on the ribose of the first base for detection by RIG-I [12]. The ligands of MDA5 and LGP2 are less understood relative to what is known of RIG-I. However, MDA5 has been reported to bind ssRNA as well as dsRNA [12,13]. Once bound to an RNA, an RLR is free to associate with the Mitochondrial antiviral signaling (MAVS) adapter protein, leading to the activation and nuclear translocation of IRF3. Taken together, activation of PRRs results in the upregulation of proinflammatory genes, including that of Interferon beta (*IFNB1*) (Figure 1) [11].

## 3. PRR Activation Induces Significant Changes to the Gene Expression Program of Cells

The PRR-dependent signaling pathways that ensue ultimately lead to profound transcriptional changes that drive a cell from a naïve to an immune-activated state, particularly upon the expression of interferon beta (IFNB, Figure 1). For example, IRF3-dependent immune activation can lead to the upregulation of almost 1500 genes and downregulation of almost 700 genes, all defined as IFN stimulated genes (ISGs) [14]. While there is no question that PRR signaling pathways and subsequent transcriptional activity are primary hallmarks of immune activation, it is important to recognize–particularly during a viral infection–that pathogenic pathways purposefully interfere with host gene expression; either through suppressing cellular transcription itself or through preventing the translation of host transcripts [15,16]. Therefore, regulation at the RNA level provides an additional opportunity for the cell to maintain post-transcriptional control of gene expression by affecting RNA maturation, localization, stability/degradation, and for mRNAs, translation to protein [5,17,18]. Out of 2367 ISGs annotated in the Interferome and 1433 RBPs annotated by Gerstberger et al., 349 proteins belong to both categories, highlighting the critical importance of post transcriptional control in the innate immune response, [5,19].

Particularly during a stress response, such as a viral infection, RNPs can form stress granules and P bodies [20]. These concentrated RNA processing centers contain a dynamic protein and RNA composition related to translation initiation and mRNA degradation, respectively [21]. While the host cell has a tightly coordinated gene expression program upon detection and in defense of a viral pathogen, viruses have evolved mechanisms of evasion and co-option of mRNA metabolism, forming somewhat of an evolutionary arms race between the two organisms. Though vRNA mimics certain molecular features of cellular mRNA to hijack cellular mRNA metabolism, including the formation and utilization of stress granules and P bodies, these disguises are imperfect [20]. Replication intermediates pose a particular vulnerability for detection by PRRs and other cellular RBPs but are transient [1,2,3].

## 4. Maturation of Cellular mRNA Ostensibly Marks Transcripts as “Self”

The maturation process of an mRNA occurs in the nucleus where it can be capped, spliced, chemically modified at internal bases, polyadenylated, and eventual exported into the cytoplasm. The nuclear addition of the 7-methylguanosine (m7G) cap occurs in three steps: (1) cleavage of the PPP of the first transcribed base to PP by a triphosphatase (TPase), (2) addition of a guanosine monophosphate by a guanylyltransferase (GTase), and (3) methylation at the N7 residue of the guanosine by RNA Methyltransferase (RNMT) (Figure 1b) [22]. The m7G cap can be further modified through methylations of the ribose 2′ hydroxyl on the first and second transcribed bases, modifications that presumably mark the mRNA as “self”. Mimicking cellular cap structures is one way in which viruses evade detection by innate immune proteins, such as RIG-I and Interferon induced protein with tetratricopeptide repeats 1 (IFIT1) [3,23,24]. While RIG-I primarily senses dsRNA, a dsRNA containing methylation at the 2′ hydroxyl of the first transcribed base (cap 1) can evade RIG-I sensing [25]. IFIT1 is a ssRNA cap-binding protein that can detect incomplete cap structures [25,26,27,28]. The binding of IFIT1 on an mRNA results in the prevention of eukaryotic initiation factors from associating with the transcript, thus preventing its translation and protein production [29]. Evasion of IFIT1, however, can be achieved through the cap 1 modification and certain secondary structures in the 5′ UTR [29,30]. Many viruses encode a 2′-O-methyltransferase in order to facilitate such escape, and some viruses, such as influenza A virus (IAV), can steal caps from host transcripts through a mechanism known as cap-snatching [31].

More details on the cap structure and the role of IFIT1 in the context of a viral infection will be discussed later in this review. Polyadenylation is achieved primarily by the activities of nuclear Poly(A) binding protein (PABPN1), Cleavage and polyadenylation specificity factor (CPSF), and Poly(A) polymerase (PAP) [32,33]. The splicing of introns also occurs co-transcriptionally and is orchestrated by numerous RNPs, collectively termed spliceosomal components [34]. Splicing and alternative assembly of transcripts through the omission or inclusion of alternate exons is a highly coordinated process that occurs in the nucleus for which entire reviews are dedicated [34,35]. Viruses can both modulate cellular alternative splicing and utilize the spliceosome for their own benefit [34,35]. The nuclear events that lead to mRNA maturation are ostensibly to mark transcripts as appropriately processed and derived as ‘safe’ for cytoplasmic translation, thus also preventing their destruction by the exosome.

## 5. Nuclear Export and mRNA Translation Are Subject to Viral Co-Option

The nuclear export of a cellular mRNA is mediated in part by an orchestration of multiple RBPs to form heterogeneous nuclear RNP C (hnRNPC) [36]. Early in maturation, hnRNPC directs the transcript towards the export pathway where the Transcription-export complex (TREX) binds the RNA and functions as an adapter between the mRNA and the heterodimeric nuclear export receptor, TAP/p15, allowing the mRNA to be shuttled through the nuclear pore complexes (Figure 1b) [37]. Once in the cytoplasm, the RNA is then subject to translation, which occurs as a tightly coordinated series of events facilitated by RNPs. The translation preinitiation complex (PIC), which consists of Eukaryotic initiation factor (eIF) proteins -2, -5, -3, and the 40S ribosome subunit attaches to an mRNA as eIF4F and eIF4B cooperate to unwind any secondary structure [38]. eIF4F is a multi-protein complex composed of eIF4E (the cap binding protein), eIF4A (the helicase), and eIF4G (a scaffold protein) [39]. eIF4F, met-tRNAi, and PABPC1 then prepare the mRNA for PIC attachment, which then forms the initiation complex. Next, a complete ribosome is formed and generates a peptide from the mRNA sequence (Figure 1b) [38,39]. While IFIT1 sequesters incompletely capped RNA from cap-dependent translation, cap-independent translation can be achieved through internal ribosome entry sites (IRES), a mechanism used by poliovirus (PV), rhinovirus, encephalomyocarditis virus, and hepatitis C virus, to name a few [40,41,42]. Interestingly, cellular transcripts can also utilize IRES mediated translation in addition to another cap-independent translation initiation mediated by m6A [40,43,44].

## 6. Pathways That Modulate RNA Stability Provide Additional Opportunities for Viral Pathogenesis

The control of mRNA stability or degradation is facilitated by several functional classes of RBPs. Most simply, mRNA can be digested by ribonucleases (RNases) belonging to two different classes: exonucleases and endonucleases [45]. These RNAses can digest an RNA at any stage within its lifecycle, particularly to eliminate aberrantly processed products such as transcripts with improper 5′ or 3′ termini, failed nuclear export, or stalled ribosomes [45]. The most common cytoplasmic RNA degradation pathway occurs via deadenylation by the Poly(A)-nuclease-2 and -3 (PAN2-PAN3) and Carbon catabolite repression-negative on TATA-less (CCR4-NOT) complexes, decapping by the DCP1-DCP2 decapping complex, and degradation by 5′-3′ Exoribonuclease 1 (XRN1) (Figure 1b) [46]. Indeed, XRN1 can be hijacked by IAV to inhibit the IFN response [47]. Additionally, microRNAs (miRNAs) and their cognate partner proteins, Argonautes 1–4 (AGOs1-4) play a role in mRNA stability [48]. AGO proteins complexed with miRNA compose the RNA-induced silencing complex (RISC), which can bind to the 3′ UTR of target transcripts in a seed sequence-dependent manner.

Complete small RNA complementarity by an AGO2-containing RISC can lead to AGO2 dependent cleavage of transcripts, whereas RISC complexes (containing any AGO paralog) that bear seed sequence-dominant complementarity leads to the recruitment of the CCR4-NOT and DCP1-DCP2 complexes, thus promoting transcript decay [48].

**Figure 2:**
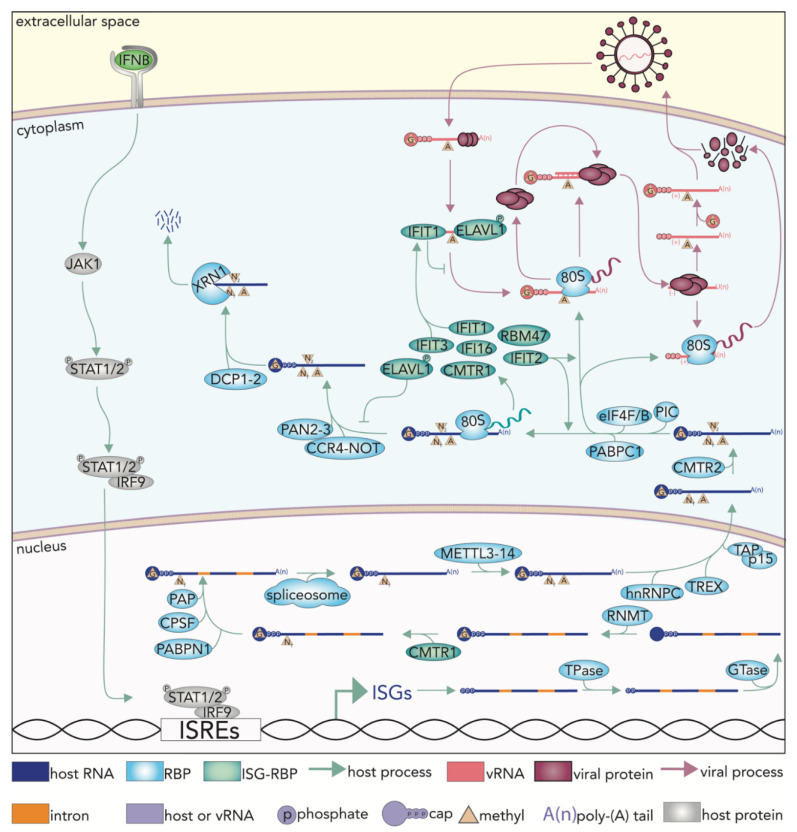
Immune stimulation and viral infection lead to a tension between host and pathogen for control of cellular RBPs, RNA metabolic processes, and gene expression. IFNB induces a JAK/STAT signal transduction event culminating in activation of IRF9 and its association with IFN stimulated response elements (ISREs) within the promoters of interferon stimulated genes (ISGs) [1,2,3]. RBPs facilitate all aspects of RNA metabolism including mRNA maturation, initiation of translation, and stability–processes which are also subject to hijacking by viral pathways. Capping and polyadenylation of mRNAs are an important step in transcript maturation and are facilitated by nuclear-localized RBPs; see text for additional details. Once a transcript is fully matured, it is exported out of the nucleus typically via the TREX complex where it can encounter additional RNA modifications; for example, methylation of the second transcribed base by CMTR2. The formation of a competent PIC is a limiting step to translation initiation and recruitment of the 80S ribosomal subunit. RNA stability in the cytoplasm can be influenced by a number of factors including the association of PAN2/3 and CCR4-NOT which leads to deadenylation, decapping (DCP1/2) and eventual degradation by XRN1. In nearly all cases, there exist viral mechanisms that can usurp control of RNA processes for their own purposes. Thus, the existence and levels of select host RBPs can impact the efficiency of viral replication. Existing and newly expressed RBPs, as a result of innate immune activation (ISG-RBPs), are collectively responsible for managing host gene expression, but also in the surveillance and clearance of pathogenic RNA. INTERFERON INDUCED PROTEIN WITH TETRATRICOPEPTIDE REPEATS 1 (IFIT1) heterodimerizes with IFIT3 and binds incompletely capped RNA to prevents translation of that RNA. IFIT2 is associated with an increase in ISG and vRNA translation [53]. Constitutively expressed protein, EMBRYONIC LETHAL VISION-LIKE PROTEIN 1 (ELAVL1), is phosphorylated during immune stimulation and primarily localizes to the cytoplasm where it binds the 3′ UTR of ISGs to stabilize them. ELAVL1, IFI16, and FASN, which can all bind cellular RNA, also bind CHIKV RNA.

While mimicry of cellular molecules is a mechanism of survival for viruses, so is the recruitment or molecular hijacking of cellular proteins. Notably for RNA viruses, their RNA genomes (or antigenomes) must be translated by cellular machinery (Figure 2). Thus, cellular RBPs are of critical importance in either preventing or promoting a viral infection. Parsing out the roles of cellular RBPs in the context of a viral infection remains an exciting challenge, particularly when considering how these RBPs’ activities are distributed between regulating host versus viral gene expression. Recent work characterizing early host-virus interactions have led to the discovery of a wealth of cellular proteins that can directly interact with vRNA—many of which were not previously annotated as having RNA binding capacity [17,49,50,51,52]. Thus, one may question whether these non-canonical RBPs have roles in cellular RNA metabolism, even under non-pathogenic states. 

## 7. Surveying vRNA-RBP Interactions in Cells Enables the Discovery and Characterization of RBPs; New and Old

To date, the identification and characterization of RBP targets are predominantly achieved as single-RBP-centric endeavors via the capture of an RBP, usually by antibodies, followed by high-throughput sequencing of its co-enriched RNAs [54,55,56,57,58,59]. To mitigate the possibility of RNP assembly post-cell lysis, crosslinking procedures were developed in which RNA and proteins would be chemically linked in living cells or tissue [60]. There are a wide variety of UV-based and chemical-based cross-linking and immunoprecipitation (CLIP) approaches, all of which rely on the use of antibodies to isolate an RBP of interest to enrich for corresponding target transcripts [59,60,61,62,63,64,65,66,67,68,69,70,71,72,73]. While the discovery and characterization of regulated transcripts of a single RBP remains an essential strategy towards building a global understanding of PTGR, an analogous rationale can be made for investigations that aim to discover and characterize the constellation of RBPs that act upon groups of related RNAs, or even a singular transcript. 

Even prior to the development of crosslinking approaches were strategies in which the goal was to identify the proteins that were bound to subsets of RNAs or single RNAs [50,51,74,75,76,77,78,79]. Early RNA-centric papers involved the use of aptamer tagged RNA, in vitro transcribed RNA, or immobilized oligo bait to capture proteins from cell lysates or protein microarrays [74,80,81,82,83,84,85,86]. These early studies revealed the potential wealth of proteins that had not previously been associated with RNA. Nonetheless, while they enabled a broader strategy for investigating RNA-bound proteomes, these methods could not account for the potential identification of nonspecific lysis-induced RNP reassembly [60]. To address this concern, several RNA capture methods, which collectively rely upon UV-crosslinking, were developed to stabilize RNPs prior to lysis [50,51]. These methods enable the isolation of photocrosslinked RNA-protein complexes via oligo(dT) capture, providing the opportunity for both interrogation of the mRNA-bound proteome and the protein occupancy on polyadenylated RNAs. These techniques have since been optimized for higher sensitivity and specificity of identified transcripts, for identifying RBPs on non-polyadenylated RNAs, and to survey changes in the global RNA-bound proteome during viral infection [52,87,88]. Other methods to recover non-polyadenylated and long noncoding RNAs (lncRNAs) have also emerged in recent years [75,89,90,91]. Notable single RNA-centric techniques were developed to discover the RNA-bound proteome of specific lncRNAs, which used hybridization-based anti-sense oligonucleotide probes to isolate RBPs on the lncRNAs of interest [75,76,77]. In surveying the individual discoveries of each of these reports, what became clear was the sheer number of unanticipated RNA-protein interactions that can exist in a cell, many by non-canonical RBPs, across a wide variety of biological contexts–suggesting a richer tapestry of proteins contributing to PTGR that were not until now accessible to investigation [17,92].

Infection by RNA viruses presents a unique biological context in which the viral genomes themselves are the RNAs of interest, particularly given their impact to human health and disease. Since vRNAs must interact with cellular proteins for infection to progress, several methods were developed to probe RBPs specifically interacting with vRNAs in live cells infected with replication-competent virions (Table 1) [93,94].

**Figure 3:**
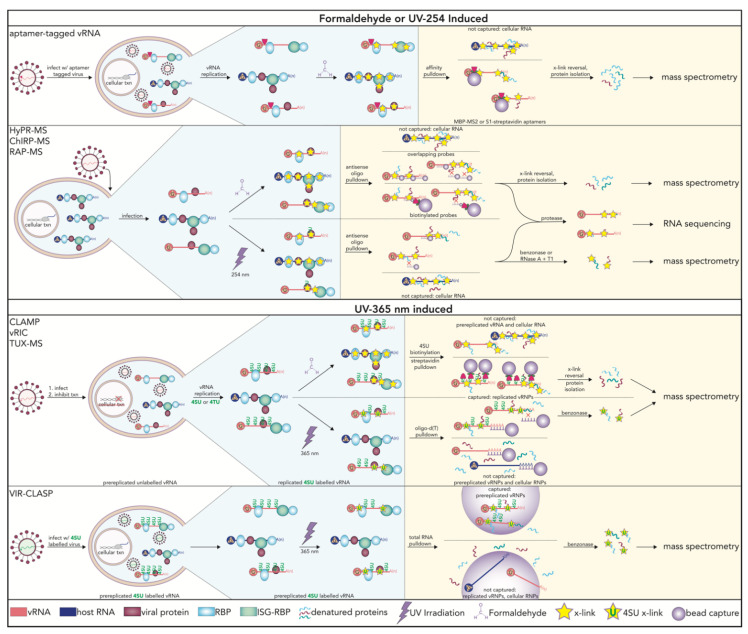
An overview of methods that interrogate vRNA-RBP interactions in live cells during viral infection. Methods to probe vRNA-RBP interactions are grouped based on overlapping methodologies. The blue shaded region denotes the cellular environment in which interactions are stabilized. The yellow shaded region shows how those interactions are captured. Also outlined in the yellow shaded region are interactions that are captured vs. those that are not. Notably, formaldehyde-based methods result in the crosslinking and capture of protein-protein interactions whereas 4-thiouridine (4SU) based methods (bottom two panels) have the highest level of stringency. vRNA: viral RNA; txn: transcription; x-link: crosslink; 4TU: 4-thiouracil; RBP: RNA binding protein; ISG: interferon stimulated gene; HyPR-MS: Hybridization Purification of RNA-protein complexes followed by Mass Spectrometry; RAP-MS: RNA Antisense Purification and quantitative Mass Spectrometry; ChIRP-MS: Comprehensive Identification of RNA binding Proteins by Mass Spectrometry; vRIC: vRNA Interactome Capture; CLAMP: Cross-Link-Assisted Messenger ribonucleoprotein Purification; TUX-MS: Thiouracil Cross-Linking Mass Spectrometry; VIR-CLASP: VIRal CrossLinking And Solid-phase Purification.

Notably, thiouracil cross-linking mass spectrometry (TUX-MS) utilizes the photoreactive ribonucleotide analogue, 4-thiouracil (4TU), along with a cellular transcription inhibitor to label only vRNAs during infection [94]. While advantageous in its ability to assess vRNA-RBP interactions in a cellular environment, this method is limited by its requirement to inhibit cellular transcription. This transcription inhibition eliminates the ability to assess global changes in vRNA-RBP interactions during the transcriptional switch from a naïve to activated state. Further, only RNA viruses that replicate independently of cellular transcriptional machinery can be interrogated. Both TUX-MS and its more quantitative iteration, qTUX-MS, require efficient hybridization between the target vRNA and DNA probes as they depend on oligo(dT) or antisense oligonucleotides for capture, respectively [102]. Other methods have also emerged to elucidate vRNA-RBP interactions in cells infected with Dengue virus (DENV), PV, human immunodeficiency virus 1 (HIV-1), Sindbis virus, rhinovirus, and the virus responsible for the recent global pandemic, severe acute respiratory coronavirus-2 [95,96,97,103]. With each new method came unique advantages as well as important considerations. Overall, however, these have led to the broadening of an investigators’ ability to dissect vRNA-host protein interactions across different virus types, as compared in Figure 3 [59,104].

Many of the techniques outlined in Table 1 and Figure 2 use oligonucleotide- and hybridization-dependent capture of the RNAs of interest. Oligonucleotide pulldowns balance the need for sufficient complementarity with mitigating the risk of co-enriching for hybridized but unintended RNAs. However, a more important consideration is the fact that bound RBPs can occlude corresponding regions within RNA and thus alter the efficiency of probe hybridization. This poses a significant—and ironic—limitation in that the proteins of interest can limit their own detection. Similar to host-encoded RNA transcripts, vRNAs are anticipated, and have been observed, to encounter a dynamic assembly of RBPs that are time- and/or condition-dependent [103]. Accordingly, sequence-dependent pulldowns will naturally be affected by varying sequence availability on the target vRNA due to changes in RBP occupancy. To address this concern, most modern strategies capture interactions under protein denaturing conditions. Nonetheless, owing to the difficulties of predicting which target sequences remain occluded and which anti-sense probes remain variably affected across conditions, this type of bias can confound accurate assessments of the true enrichment levels of identified RBPs relative to each recovered protein. In particular, it would be difficult to define the differential enrichment of recovered RBPs from time-resolved or condition-dependent experiments. Towards addressing these challenges, viral crosslinking and solid phase purification (VIR-CLASP) was explicitly developed to bypass sequence-dependent purification of vRNA-RBP complexes through total RNA capture of crosslinked proteins [103]. Given that VIR-CLASP does not rely on sequence-based capture, it is applicable to virtually all RNA viruses and was confirmed to capture vRNA-RBP interactions from representative viruses of the following families: *Togaviridae, Orthomyxoviridae, Picornaviridae, Coronaviridae, Flaviviridae, Rhabdoviridae*, and *Phenuiviridae* [103]. Additionally, specific incorporation of 4-thiouridine (4SU) within the incoming viral genome and subsequent infection of unlabeled cells, allows for interrogations of interactions solely with the pre-replicated viral genome [103]. Interestingly, 50% of the CHIKV interactome proteins identified are ISGs and 40% of the interactome proteins identified are novel RBPs [103]. Recently, vRNA interactome capture (vRIC), a method for identifying vRBPs later in the viral lifecycle was developed [96]. vRIC involves cellular RNA polymerase II (RNAPII) inhibition of virus infected cells followed by treatment with 4SU [96]. Similar to TUX-MS, RNAPII inhibition leads to 4SU incorporation into vRNA and not cellular RNA [96]. This allows for specific crosslinking of newly synthesized vRNA transcripts, rather than the incoming genome, followed by oligo(dT) pull down of vRNA-RBP complexes and subsequent proteomic analysis [96].

Though most of the techniques in Table 1 and Figure 2 were performed on single stranded, positive sense viral genomes, VIR-CLASP was applied to a wider range of viruses, including those with negative sense and segmented genomes [103]. Considerations for choosing a certain technique for a virus of interest include genomic structure, replication cycle, and cellular tropism, with additional criteria recently reviewed [59,105]. Covalent crosslinks are introduced via formaldehyde or UV irradiation. While UV irradiation techniques are the most selective, they can be biased by transcript sequence and amino acid composition of bound proteins. Formaldehyde crosslinking can result in the capture of protein-protein interactions, thus limiting associated techniques from discovering novel RBPs (Figure 2). However, formaldehyde is useful for the capture of larger RNPs. RNA can be isolated through aptamer tags, antisense oligonucleotides, or nonspecific pull-down. Through strong affinity to interactors, aptamer tags have high stringency. However, aptamer tags can interfere with RNA structure, prevent RBP binding, and yield nonfunctional virions, though methods for post-lysis addition of the tags do exist [106]. 

Taken together, both RNA- and protein-centric methodologies can be employed to investigate the rapidly evolving transcriptional landscape induced by a viral infection. What influences the changing binding and functional landscape of these RBPs? To explore this question, we will discuss recent findings reporting on the expanded roles of specific RBPs that were revealed by examining their behaviors during viral pathogenesis and/or immune stimulation. We will focus on case studies that delve into the recognition of RNA 5′ cap structures, m6A modifications, the emerging activities of select noncanonical RBPs, and RNA stability. These and other contemporary reports prompt a re-examination of whether an RNA can be neatly designated as “self” or “foreign”.

## 8. RNA Recognition Predisposes Fate

**Figure 4:**
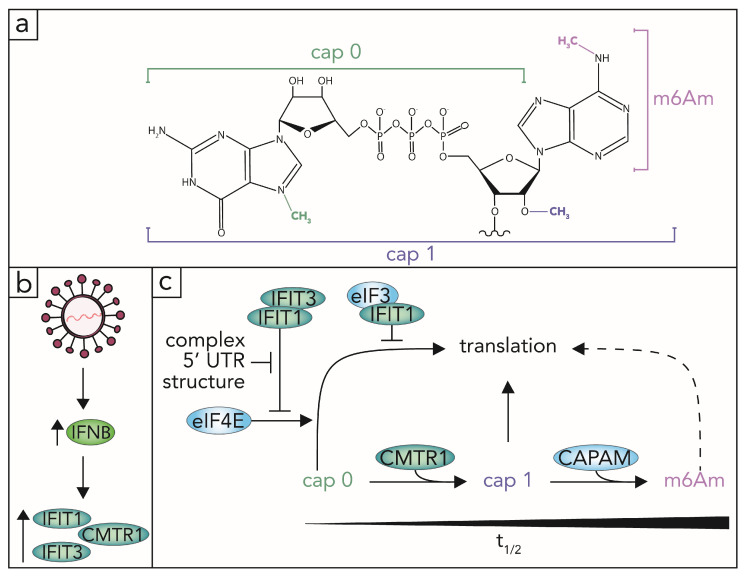
Cap modifications impact protein binding on an mRNA, thus affecting its ultimate fate. (**a**) The 7-methylguanosine cap (m7G) cap 0 structure is composed of an inverted guanosine residue that is methylated at the N7 position (green) and connected to the first transcribed base by a triphosphorylated linkage. A methylation at the 2′ hydroxyl on the ribose sugar of the first transcribed base (blue) forms the cap 1 structure. A large proportion of eukaryotic mRNA also contains a methyl-6-adenosine (m6A) residue as the first transcribed cap 1 base, denoted as 2′O-dimethyladenosine (m6Am, purple). (**b**) Upon infection with a virus, the interferon beta (IFNB) pathway is induced, resulting in the upregulation of Cap methyltransferase 1 (CMTR1) and the expression of Interferon induced protein with tetratricopeptide repeats (IFITs)- 1 and 3. (**c**) The cap 1 modification, which is co-transcriptionally added by CMTR1, is efficiently translated and bypasses IFIT1-mediated translational restriction. IFIT1 restricts translation by sequestering RNA from Eukaryotic initiation factor 4 E (eIF4E). This restriction is enhanced by heterodimerization of IFIT1 with IFIT3. A predicted stem loop structure on the 5′ UTR of cap 0 transcripts was shown to overcome translational restriction via IFIT1 [25]. IFIT1 also prevents translation through protein-protein interactions with eIF3. Whether the m6Am modification increases, decreases, or has no effect on translation is unclear, as denoted by the dashed line. However, the m6Am modification has been associated with an increase in transcript stability, as denoted by the longer half-life (t_1/2_) whereas the cap 0 modification is associated with a decrease in transcript stability.

The timing, extent, and nature of modifications on a viral transcript have a profound effect on whether the vRNA is predominantly sensed by antiviral proteins or whether it can sufficiently engage with proviral cellular machinery to continue its replication program. One major hotspot for recognition is at the 5′ end of RNA and the type of structural and chemical modifications it bears. Host-encoded mRNAs are typically co-transcriptionally modified with an m7G cap, as previously detailed. This co-transcriptional process yields a “cap 0” structure, which is defined as the 5′-to-5′ oriented addition of guanosine and its methylation at the N7 position of the base (Figure 4a). Following the formation of the cap 0 structure, an mRNA can be further methylated at the 2′ hydroxyl of the ribose sugar of the first transcribed base by Cap methyltransferase 1 (CMTR1) to form a “cap 1” structure (Figure 4a). The CMTR1-mediated cap 1 modification has historically been coined a marker of “self” RNA, although a recent study suggests that up to 12% of eukaryotic RNA leads with the cap 0 structure [107,108]. mRNA transcripts can also be methylated at the 2′-hydroxyl of the ribose sugar of the second transcribed base by Cap methyltransferase 2 (CMTR2), forming the “cap 2” structure [109,110]. While the exact purpose of the cap 2 modification is still not clear, a potential function is to mark RNA as “self”, thus preventing the binding of antiviral proteins IFIT1 and RIG-I [27,111].

The expression levels of CMTR1 (which was first characterized as “ISG95”) are sensitive to IFN stimulation (Figure 4b) [112,113]. Recently, CMTR1 was found to be required for the enhanced expression of other ISGs [114]. As up to 12% of cellular RNA may contain a cap 0 structure and the enzyme responsible for depositing the cap 1 methylation is an ISG, cap dynamics during immune stimulation may be an underexplored means of translational control. The distinction between cap 0 and cap 1 is important when considering the IFIT family of proteins. Like CMTR1, the IFITs are IFN-induced cap-interacting proteins (Figure 4b). Unlike CMTR1, the IFITs do not possess known enzymatic activity and are typically undetectable under basal conditions [115]. In humans, there are four members of the IFIT family; IFIT1, IFIT2, IFIT3, and IFIT5 [115]. The IFITs are named for their tetratricopeptide repeat motifs, a class of motif more classically implicated in protein-protein interactions [116]. Decades after their discovery, the IFITs were characterized for their RNA binding activity [117]. Since their discovery as RBPs, the IFITs have conventionally been accepted as antiviral proteins that bind incomplete vRNA cap moieties (such as cap 0), thus restricting the translation of corresponding transcripts [25,26]. In the prevailing model of translational restriction, IFIT1 prevents binding of eIF4E onto vRNA, thus preventing cap-dependent translation (Figure 4c) [26]. This can be enhanced by heterodimerization between IFIT1 and IFIT2 or IFIT3 [118]. Further, IFIT1 can also participate in protein-protein interactions with eIF3 to inhibit its binding to the mRNA (Figure 4c) [119].

Recent publications have demonstrated that the IFITs are capable of binding cellular RNA in addition to vRNA [114,120,121]. This is shifting the perspective of their roles as antiviral effectors to antiviral effectors that also regulate cellular PTGR in innate immunity. While the cap modification is not a sufficiently distinct marker between vRNA and cellular RNA for IFIT detection, the exact distinguishing factor is still to be discovered. 5′ UTR character of certain IFIT1-regulated cellular transcripts is at least partially responsible for their detection and translational restriction via IFIT1 (Figure 4c) [114]. Indeed, a region predicted to contain a stem-loop secondary structure in the 5′ UTR of several alphaviruses allows vRNA escape of IFIT1 despite the vRNA containing a cap 0 structure [29]. IFIT selective detection of cellular transcripts could serve a role in suppressing the translation of bound transcripts without affecting the mRNA stability [114,121]. Indeed, the half-life of IFIT1 in the absence of IFIT3 is as short as 1.9 h [122]. A comparison of the IFIT half-lives on an mRNA versus that of the bound mRNA has, to our knowledge, not yet been explored. In contrast to its accepted role as an antiviral effector, IFIT2 has recently been demonstrated as proviral during IAV infection [120]. In this study, IFIT2 bound both viral and cellular RNA at AU-rich regions [120]. Of the cellular transcripts bound by IFIT2, ISGs were prominent [120]. The data presented in this study suggest that IFIT2 works to increase translation of cellular mRNAs (particularly antiviral RNAs) and that IAV co-opts this activity to facilitate its replication [120].

As mentioned, a subset of eukaryotic RNAs lead with the cap 0 structure [113,114]. Strikingly, in one study, all of the detectable cap 0 modifications found in vertebrate mRNA contained m6A as the first transcribed base [113]. A cap 1 moiety with m6A as the first transcribed base is distinct from its internal counterpart and thus has its own designation: 2′-O-dimethyladenosine (m6Am, Figure 4a) [123]. Although the m6Am modification and a partial purification of the enzyme responsible for the modification were discovered in the 1970s, the enzyme responsible for depositing the N6-adenosine methylation of m6Am had only recently been discovered [123,124,125,126,127]. The methylating enzyme, Cap-specific adenosine N6-methyltransferase (CAPAM, formerly Phosphorylated-CTD interacting factor 1 (PCIF1)) is exclusive to the m6Am modification and only found in higher eukaryotes [124,125,126]. One study that discovered CAPAM as the sole m6Am methyltransferase found that 92% of mRNAs leading with adenosine as the first transcribed base contain the m6Am modification [124].

There is conflicting evidence on the functional outcome of the m6Am modification on a transcript. Separate studies have found the modification to either enhance translation, decrease translation, or have no effect on translation (Figure 4c) [118,119,120,128,129]. Studies in favor of m6Am-enhanced translation include two separate ribosome profiling studies [124,128]. One surveyed the translational efficiency of m6Am leading transcripts and found that those transcripts have a higher translational efficiency than cap 1 transcripts beginning with any of the other four bases (A, C, G, U) [128]. The other utilized CAPAM knock-out (KO) cells to find that m6Am-associated translational upregulation occurred independent of eIF4E binding activity [124]. Studies that favor m6Am-associated decreases in translation include those that used reporter transcripts and a mass spectrometric approach [53]. Reporter transcripts beginning with either cap 1-A or m6Am indicated that the m6Am mark was associated with a suppression of cap-dependent translation [53]. The mass spectrometric approach found that 505 proteins were upregulated in CAPAM KO cells vs. 17 downregulated proteins [53]. Studies that found no detectable difference in translation as mediated by CAPAM include another ribosome profiling experiment and vesicular stomatitis virus (VSV) reporter assays [125,129]. These conflicting results could be due to variation in experimental design, filtering processes used for sequencing data, cell lines used, and conditions under which the experiments were performed. Additionally, while direct, methods of probing translation using exogenous RNA expression and in vitro assays are limited by their use of one specific transcript species. The use of a single RNA species eliminates the ability to survey the complexity found in a heterogeneous cellular transcriptome. Further, other factors that can affect translation such as structure and cooperative RBP binding are difficult to control. More work will need to be done to conclusively determine the translational impact of the m6Am modification under different conditions (particularly viral infection) and in different cell types. Beyond its impact on translation, the m6Am modification also has a role in transcript stability. 

When comparing the stability of transcripts beginning with m6Am and any of the other four cap 1-modified nucleotides, m6Am-leading transcripts exhibit a longer half-life (Figure 4c) [128]. One study found that m6Am is found on highly stable and abundant transcripts, although CAPAM KO did not affect the stability of those transcripts [125]. In contrast, less stable and abundant mRNAs exhibited a steep drop in their stability with CAPAM KO [125]. In vivo stabilization of m6Am mediated transcripts has also been demonstrated in mice [124]. The stability of m6Am capped transcripts is at least in part via protection against decapping protein, DCP2 and miRNA-mediated degradation [128]. This trend in enhanced stability is not universal, though. VSV RNA stability is unaffected by the presence of the m6Am modification [129]. There are also disagreements on the dynamics of m6Am in transcript stability, and in particular the role of m6A eraser protein, FTO. While FTO could target and destabilize the 5′ localized m6Am, it is possible that the impact of FTO on transcript stability could be limited to internal m6A moieties [128,130,131].

The intersection between the m6Am modification, a cap 0 structure with m6A as the first transcribed base, and the IFITs is still a mystery. It is known that DENV (a virus that is not restricted by IFIT1) contains m6Am [107]. VSV, rabies virus, and measles virus are also known to contain the m6Am modification as catalyzed via CAPAM [123]. Interestingly, the m6Am modification does not have a detectable effect on VSV replication or gene expression [123]. Some DNA viruses including adenovirus, simian virus 40, herpes simplex virus 1 (HSV-1), polyomaviruses, and vaccinia virus also contain m6Am [132,133,134,135,136,137]. Whether m6Am exists on other viral transcripts is yet to be conclusively established [138,139]. CAPAM KO cell lines show normal growth rates under basal conditions, however under oxidative stress, they exhibit defective growth [124]. Whether CAPAM is implicated in proper innate immune functioning remains an intriguing, underexplored avenue, especially since the m6Am modification is implicated in translation of associated transcripts and mRNA stability [124,128,131]. A recent study found that CAPAM can attenuate the IFNB antiviral response during IFNB-primed VSV infection, and that this phenomenon is not influenced by decreased binding efficiency of IFIT1/3 or RIG-I to the vRNA [129]. More work will need to be done to establish potential overlapping roles of CMTR1, CAPAM, and the IFITs in cellular PTGR during innate immune activation.

While more well-studied than m6Am, the roles of internal m6A modifications in viral infection and innate immunity are complex. We will provide a brief commentary on m6A in viral infection but direct the reader to recent reviews for a more in-depth discussion on the subject [138,139]. Originally identified in the 1970’s, the methylation of internal adenosines at the N6 position (m6A) is facilitated by Methyltransferase-like proteins-3 and -14 (METTLs -3 and -4) [138,140,141]. There are currently two known m6A erasers: FTO and ALKBH5 [138]. Among the RBPs that read the m6A modification are the YTH domain containing (YTHDF) family of proteins [138,139]. As one of the most abundant mRNA modifications, m6A is implicated in multiple steps of RNA metabolism including but not limited to: maturation, localization, stability, translation, and structure [138]. In fact, each YTHDF protein plays a distinct role in the mRNA lifecycle. YTHDF1 is implicated in an increase in translation, YTHDF2 in mRNA degradation, and YTHDF3 in both processes [142,143,144,145]. Important for the scope of this review, emerging research hints that the m6A modification plays a role in both viral pathogenesis and the cellular innate immune response [138,139]. Indeed, it has been suggested that m6A may be a marker of “self” exploited by viruses to mimic host transcripts [146]. A few explanations exist for how m6A may mark RNA as self. m6A has been shown to suppress PRR activation [138,147,148,149,150]. This phenomenon can be in part explained by the m6A-mediated abrogation of dsRNA, the ligand of RIG-I [146,151]. Another potential explanation for evasion of RIG-I sensing is via occlusion by m6A reader proteins [149,152]. Whether internal m6A modifications on vRNA are considered proviral, antiviral, or neutral is dependent on the virus type, stage of the lifecycle that the virus is in, and the model cell line used [138]. For example, m6A has an antiviral effect on viruses in the *Flaviviridae* family but has a proviral effect on IAV [138].

METTL3 and METTL14 typically deposit m6A cotranscriptionally on adenosine residues within the “DRACH” motif (D = G, A, or U; R = G or A; H = U, C, or A) [138]. However, a complete understanding of parameters for target selection of the m6A modification are not known—particularly because methods to detect the modification are limited in their abilities to probe dynamic states, such as that during a viral infection or immune stimulation [138]. This poses a unique challenge in that many ISGs are RBPs [14]. Therefore, more m6A-interacting RBPs may exist than the field has currently identified [103,138,139]. Since the m6A writers are largely considered nuclear proteins, it is also an enigma how cytosol-restricted viruses obtain the m6A modifications (there is no evidence that viruses encode m6A-methylases) [138]. It has recently been shown that METTL3 and METTL14 can be shuttled into the cytoplasm in enterovirus 71 infection, VSV infection, and HSV-1 infection [153,154,155]. Further, in VSV infection, METTL3 shuttling into the cytoplasm is also associated with an attenuated IFNB-mediated immune response [155]. This serves as an example of how viruses can facilitate both their replication and immune escape. By disrupting the localization of m6A writer proteins, a virus can also dysregulate cellular PTGR. It will be interesting to see what other viruses are capable of differentially localizing the m6A writer proteins and whether this phenomenon can occur in the absence of immune stimulation. Nonetheless, addition of the internal m6A modification seems to be a dynamic process that is implicated in controlling ISG translation during an IFN response [156].

Many immune signaling transcripts, including *IFNB1,* contain m6A in their 3′UTR [138,157,158]. Yet, a portion of these transcripts lose this mark during immune stimulation, thus restricting them into the nucleus and preventing their translation, presumably to ensure an acute immune response [138,157,158]. Indeed, the m6A modification on *IFNB1* has been shown to destabilize the transcript, thus ensuring a punctuated immune response [157]. Depletion of METTL3 and YTHDF2 has also been shown to lead to an elevated induction of ISGs [157]. m6A can have other direct and indirect effects on ISG PTGR. For example, YTHDF3 mediates increases in the translation of FOXO3, an ISG transcriptional repressor [159]. Additionally, YTHDF1 has recently been shown to promote the translation of a subset of m6A-modified antiviral ISGs [156]. While YTHDF1 can promote the translation of certain ISGs, it is also implicated in the prevention of aberrant interferon signaling. It has recently been shown that the *Adenosine deaminase acting on RNA 1* (*ADAR1*) gene is m6A modified [160]. The ADAR family of enzymes are RBPs that specifically bind dsRNA [161]. ADAR1 and ADAR2 each catalyze the editing of adenosine to inosine, thus abrogating dsRNA formation, amongst other things [161,162]. The activities of ADAR1 and ADAR2 are important for regulation of the IFN response, specifically through preventing aberrant signaling resulting from the detection of endogenous dsRNA [163,164,165]. YTHDF1 enhances induction of the m6A-modified, interferon-inducible isoform of ADAR1, ADAR1P150 [166]. This enhanced induction of ADAR1P150 in turn enhances the A-I editing of certain ISGs during the IFN response. Thus, the ISGs cannot activate MDA5 and the downstream IFN response [166]. 

The YTHDF proteins also have potential effects on viral replication independent of innate immune PTGR. For example, YTHDF1-3 bind the HIV-1 genome and play an antiviral role in HIV-1 infection [167,168]. Furthermore, under stringent mass spectrometric cut-off conditions in VIR-CLASP, YTHDF2 and YTHDF3 directly interact with the pre-replicated CHIKV genome [103]. In the same study, YTHDF1 was also identified as an interactor via western blot analysis [103]. Ultimately, YTHDF1 and YTHDF3 have antiviral roles during CHIKV infection whereas YTHDF2 has a proviral role [103]. However, whether these observations were a direct consequence of YTHDF-vRNA binding remains to be explored [103].

It has recently been shown that the demethylase activity of ALKBH5 is impaired during VSV infection and that the pathways most affected by the impaired demethylase activity are metabolic pathways [169]. This study provided the first evidence on the function of a particular metabolic enzyme, Oxoglutarate dehydrogenase (OGDH), in a viral infection. By impairing the demethylase activity of ALKBH5, host cells can increase YTHDF2-mediated mRNA decay, thus resulting in reduced expression of associated proteins, particularly those (such as OGDH) that are proviral. Indeed, metabolic enzymes are often co-opted by viruses in order to generate the metabolites needed to facilitate infection [122]. Through RNA-centric approaches to identify RBPs, metabolic enzymes have emerged as a subset of noncanonical RBPs that are now able to be characterized for their moonlighting roles in RNA metabolism [170]. One such RBP, Fatty acid synthase (FASN), had both been previously characterized as a regulator of viral replication and also discovered as an RBP in early RNA interactome screens [50,51]. FASN was then shown to directly bind both viral and cellular RNA via VIR-CLASP and photoactivatable ribonucleoside analogue-CLIP, respectively [103]. Interestingly, while enzymatic activity was required for regulation of vRNA levels, it was not required for regulation of viral protein levels—even as the physical presence of the enzyme still was [103]. This bears an important question: is a particular RNA regulated by RBPs or is the RNA itself a regulator of RBPs? For the scope of this review, one may especially wonder whether a particular RNA can serve a regulatory role during viral infection [171,172]. This conundrum is highlighted by lncRNAs, which are enigmas amongst the RNA world for their lack of protein coding potential. With no potential to make a protein, one may wonder why the cell spends resources to transcribe these RNAs. Like their protein-coding counterparts, some lncRNAs are stimulated by viral infection and IFN [173,174]. lncRNA-ACOD1, in particular, is activated via the NF-κB pathway in response to a variety of different viral infections [174]. lncRNA-ACOD1 has been described as a proviral RNA that binds the metabolic enzyme, Glutamic-oxaloacetic transaminase 2 (GOT2) [174]. lncRNA-ACOD1 binds GOT2 in such a way that enhances the catalytic activity and metabolic output of GOT2, thus assisting in viral pathogenesis [174]. It is important to note that metabolic enzymes are not the only noncanonical RBPs undergoing investigation. IFN gamma inducible protein 16 (IFI16), an ISG and conventionally accepted PRR of pathogen-associated DNA, has recently been demonstrated to have RNA binding activity [103]. During CHIKV infection, IFI16 is an antiviral protein that restricts viral replication independent of its well characterized role of transcriptional regulation [103]. During IAV infection, IFI16 transcriptionally upregulates RIG-I and also directly binds both vRNA and RIG-I, collectively increasing the sensitivity of RIG-I signaling. Ultimately, characterizing these novel RNA-RBP interactions can lead to the discovery of novel regulators of viral infection, thus providing additional avenues for research on antiviral treatments.

**Figure 5:**
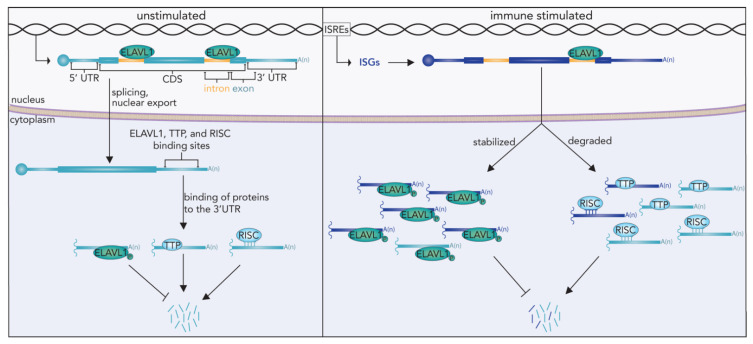
ELAVL1, TTP, and RISC compete for AREs on the 3′ UTR of mRNA. Nuclear ELAVL1 binds to intronic regions of an mRNA to assist in proper splicing. Upon immune stimulation and subsequent expression of interferon stimulated genes (ISGs), cytoplasmic ELAVL1 is phosphorylated and preferentially binds AU rich regions (AREs) within the 3′ untranslated region (UTR) of ISGs, thus enhancing the stability of the transcripts. AREs of the 3′ UTR can also be bound by the RNA-induced silencing complex (RISC) or TRISTETRAPROLIN (TTP), both of which are implicated in degradation of the RNA. The 3’ UTRs bound by ELAVL1, TTP, or RISC are represented as abbreviated sections of full-length RNA transcripts.

While noncanonical RBPs reveal exciting new horizons for investigation, canonical RBPs and their differential impacts during immune activation are not to be overlooked. Indeed, many canonical RBPs have been characterized based on their abilities to modulate the stabilities of target transcripts. For example, Embryonic lethal abnormal vision-like protein 1 (ELAVL1), which has been shown to bind m6A, typically binds to AREs within the 3′ UTRs and introns of target transcripts [14,175,176,177,178,179,180]. These same cis-elements are often flanked or juxtaposed by sequences that can be recognized by the aforementioned RISC complex [48]. Furthermore, proteins such as Tristetraprolin (TTP), T cell-restricted intracellular antigen 1, and Fragile X-related protein 1 facilitate the decay of transcripts, including immune related transcripts [181,182,183,184]. TTP, in particular, also binds AREs and recruits the CCR4-NOT complex for degradation of mRNA [185,186]. The binding of ELAVL1 on cis-elements within mRNAs can prevent RISC or TTP from binding, thus sparing associated transcripts from degradation and increasing their net half-lives (Figure 5) [187]. Recent work has shown an essential role for ELAVL1 in extending the transcript half-lives of ISGs during IRF3 dependent immune stimulation of cells, thus presumably allowing for a more durable innate immune response [14]. RNA-binding motif-containing protein 47 (RBM47) has also recently been discovered to stabilize mRNA during innate immune stimulation [188]. RBM47 is an ISG that stabilizes IFN alpha/beta receptor 1 mRNA during RNA virus infection as a mechanism to maintain an immune stimulated state [188].

It is likely that ISG RBPs did not evolve strictly to manage pathogen invasion—but to address cellular stressors more broadly. Discovering the role of their cellular target spectra is a challenge invigorated by the methodologies and reports discussed in this review. Areas of renewed interest highlighted here include RBPs that bind 5′ cap structures and m6A modifications. Both noncanonical RBPs and those involved in RNA stability are a subset of RBPs whose roles are being uncovered by studies on viral infection and immune activation. While an in-depth characterization of RBPs during viral infection can reveal novel functionalities restricted to the metabolism of vRNA, it can also give insight into an RBP’s native physiologic function in the absence of infection. As the distinction between hallmarks of “host” vs. “viral” RNA are becoming muddled, the lines between what have classically been defined as “host-specific RBPs” and “viral specific RBPs” are also blurred. In turn, strictly defining an RBP as either a pro- or anti-viral factor has become an increasingly difficult task. Perhaps these distinctions have been assigned through observed opportunity costs regulated by the stoichiometry of an RBP relative to available RNA substrates and the simplest explanation is true—RBPs cannot be neatly categorized into two groups.

## Figures and Tables

**Table 1:** RNA-centric approaches to capture vRNA-RBP interactions from live cells have overlapping yet distinct methodologies. Outlined are crosslinking agents, methods of capture, viruses probed, advantages, and disadvantages of each technique.

Method	Viruses Probed	Genome Type	Method of X-Linking	Method of Capture	Advantages	Disadvantages
aptamer tagged vRNA *	PV [95]	(+) ssRNA	n/a	aptamer tagged vRNA	strand-specific RNA pulldown; strong aptamer-ligand interactions; applicable to any stage of viral lifecycle	no x-linking; high noise levels; difficult to generate replication-competent virus; tag can affect RNA structure and/or prevent RBP binding
CLAMP	SINV [96]	(+) ssRNA	formaldehyde	4SU biotinylation, affinity pulldown	captures transient and protein-protein interactions; strong affinity interactions allow high stringency; applicable to any stage of viral lifecycle	inhibits cellular txn; virus must replicate independent of cellular txn; nonspecifically x-links all macromolecules, can isolate indirect interactions
HyPR-MS	HIV-1 [97]	(+) ssRNA	formaldehyde	antisense oligos	no manipulation of vRNA; captures transient and protein-protein interactions; applicable to any stage of viral lifecycle; adaptable to splice variants [98]	nonspecifically x-links all macromolecules, can isolate indirect interactions; oligos can anneal to cellular RNA; RBPs can prevent annealing of oligos
ChIRP-MS	DENV, ZIKV, RV, SARS-CoV-2	(+) ssRNA	formaldehyde	tiling antisense oligos	captures transient and protein-protein interactions; tiling oligos enable full length vRNA capture; applicable to any stage of viral lifecycle	nonspecifically x-links all macromolecules, can isolate indirect interactions; oligos can anneal to cellular RNA; RBPs can prevent oligo annealing
RAP-MS	SARS-CoV-2 [99]	(+) ssRNA	UV254 nm	antisense oligos	x-links only nucleic acid-bound proteins; oligos can be customized to virus of interest; applicable to any stage of viral lifecycle	UV254 is less stringent than UV365; oligos can anneal to cellular RNA; RBPs can prevent oligo annealing; interactions cannot be designated to a specific stage of viral lifecycle
x-linking and antisense purification *	DENV [100]	(+) ssRNA	UV254 nm	antisense oligos	x-links only nucleic acid-bound proteins; oligos can be customized to virus of interest; applicable to any stage of viral lifecycle	UV254 is less stringent than UV365; oligos can anneal to cellular RNA; RBPs can prevent oligo annealing; interactions cannot be designated to a specific stage of viral lifecycle
vRIC	SARS-CoV-2, SINV [96]	(+) ssRNA	UV365 nm	oligo-d(T)	labels only vRNA; x-links only 4SU-bound proteins; captures replicated interactions	inhibits cellular txn; virus must replicate independent of cellular txn; requires poly(A) vRNA; interactions cannot be designated to a specific stage of viral lifecycle
TUX-MS	PV [101]	(+) ssRNA	UV365 nm	oligo-d(T)	labels only vRNA; x-links only 4SU-bound proteins; captures replicated interactions	requires UPTR expression to convert 4TU to 4SU; inhibits cellular txn; virus must replicate independent of cellular txn; requires poly(A) vRNA; interactions cannot be designated to a specific stage of viral lifecycle
qTUX-MS	DENV [102]	(+) ssRNA	UV365 nm	antisense oligos	x-links only 4SU-bound proteins; oligos can be customized to virus of interest; captures replicated interactions; quantitates relative protein amounts	requires UPTR expression; SILAC is not applicable to all systems; labels both viral and cellular RNA; oligos can anneal to cellular RNA; RBPs can prevent oligo annealing; interactions cannot be designated to a specific stage of viral lifecycle
VIR-CLASP	CHIKV, IAV, EMCV **, MHV **, ZIKV **, VSV **, RVFV ** [103]	(+/-) ssRNA, segmented (-) ssRNA	UV365 nm	nonspecific RNA pulldown	vRNA in virion is labelled; x-links only 4SU-bound proteins; nonspecific pulldown minimizes bias; captures pre-replicated interactions	cannot capture replicated interactions; 4SU incorporation into vRNA requires optimization

(q)TUX-MS: (quantitative) Thiouracil Cross-Linking Mass Spectrometry; HyPR-MS: Hybridization Purification of RNA-protein complexes followed by Mass Spectrometry; CLAMP: Cross-Link-Assisted Messenger ribonucleoprotein Purification; ChIRP-MS: Comprehensive Identification of RNA binding Proteins by Mass Spectrometry; VIR-CLASP: VIRal CrossLinking And Solid-phase Purification; RAP-MS: RNA Antisense Purification and quantitative Mass Spectrometry; vRIC: vRNA Interactome Capture; x-link: crosslink; SARS-CoV-2: Severe Acute Respiratory Syndrome Coronavirus 2; PV: poliovirus; DENV: Dengue Virus; HIV-1: human immunodeficiency virus-1; SINV: Sindbis virus; ZIKV: Zika Virus; RV: rhinovirus; CHIKV: Chikungunya virus; IAV: influenza A virus; EMCV: encephalomyocarditis virus; MHV: mouse hepatitis virus; VSV: vesicular stomatitis virus; RVFV: Rift Valley fever virus; (+)ssRNA: positive sense, single stranded RNA; (-)ssRNA: negative sense, single stranded RNA; dsRNA: double stranded RNA; vRNA: viral RNA; 4TU: 4-thiouracil; 4SU: 4-thiouridine; UPTR: uracil phosphoribosyltransferase; SILAC: Stable Isotope Labelling by Amino acids in Cell culture; txn: transcription. * Method does not have an official name, ** Viruses used as proof-of-concept without in-depth analysis.
